# The interaction of Notch and Wnt signaling pathways in vertebrate regeneration

**DOI:** 10.1186/s13619-020-00072-2

**Published:** 2021-04-01

**Authors:** Junying Gao, Lixia Fan, Long Zhao, Ying Su

**Affiliations:** 1 Institute of Evolution & Marine Biodiversity, Ocean University of China, Qingdao, 266003 Shandong China; 2 College of Fisheries, Ocean University of China, Qingdao, 266003 Shandong China; 3 College of Marine Life Sciences, Ocean University of China, Qingdao, 266003 Shandong China

**Keywords:** Regeneration, Notch signaling, Wnt signaling, Crosstalk

## Abstract

Regeneration is an evolutionarily conserved process in animal kingdoms, however, the regenerative capacities differ from species and organ/tissues. Mammals possess very limited regenerative potential to replace damaged organs, whereas non-mammalian species usually have impressive abilities to regenerate organs. The regeneration process requires proper spatiotemporal regulation from key signaling pathways. The canonical Notch and Wnt signaling pathways, two fundamental signals guiding animal development, have been demonstrated to play significant roles in the regeneration of vertebrates. In recent years, increasing evidence has implicated the cross-talking between Notch and Wnt signals during organ regeneration. In this review, we summarize the roles of Notch signaling and Wnt signaling during several representative organ regenerative events, emphasizing the functions and molecular bases of their interplay in these processes, shedding light on utilizing these two signaling pathways to enhance regeneration in mammals and design legitimate therapeutic strategies.

## Background

Regeneration is a complex biological process, by which the tissue/organs restore their structures and functions after injury (Poss 2010). Regeneration occurs widely in animals, although their regenerative capacities vary among species. Invertebrates, such as planarians and Hydra, can regenerate the entire body. Most vertebrates possess prominent capacities to regenerate damaged structures at the embryonic/larval stages, but this ability plummets as development proceeds. Many adult vertebrates, except mammals, still display spectacular capacities to fully regenerate certain organs or appendages after injury. In contrast, the regenerative capacity in adult mammals is generally limited within the liver, bones, skeletal muscle, and skin to some degree, whereas other organs/tissues, like heart and hair cells, fail to structurally or functionally recover after injury (Brockes and Kumar 2008; González-Rosa et al. 2017; Knopf et al. 2011; Poss et al. 2003; Tu and Johnson 2011). Based on these interspecies differences, it is believed that the regeneration capacity is ancestral although it has occasionally degenerated during the evolution of vertebrates (Wagner and Misof 1992).

During post-injury repair, tissue-specific stem cells (or progenitor cells), which are typically quiescent in healthy condition, are activated to undergo proliferation and differentiation in order to maintain tissue homeostasis. Alternatively, some regenerative systems utilize the existing mature cells to generate new structures by triggering them de-differentiation into multipotent progenitor cells or directly trans-differentiation (Jopling et al. 2011; Kolios and Moodley 2013; Leeman et al. 2014; Potten and Loeffler 1990). Accordingly, the regeneration process requires the concerted actions of multiple regulatory mechanisms controlling cell proliferation, differentiation, patterning, and/or de-differentiation, trans-differentiation.

Cellular signaling is one of the fundamental mechanisms to regulate cell behaviors. Cells can respond to a variety of intracellular and extracellular signals. Many important signaling pathways, which are active during embryonic development, are activated again during organ regeneration. Notch signaling and Wnt/β-catenin signaling are two such pathways, playing critical roles during both animal development and regeneration. These two signaling pathways are evolutionarily conserved and involved in numerous cellular processes. Furthermore, Notch and Wnt signaling pathways frequently collaborate to regulate plentiful biological events. The interaction between these two pathways has been extensively investigated in regenerative tissue/organs (Fre et al. 2009; Jayasena et al. 2008; Kim et al. 2012; Li et al. 2011a; Li et al. 2015; Pannequin et al. 2009). In this review, focusing on the vertebrate models, we summarize the regenerative roles of Notch and Wnt signals in several well-established organ regeneration systems, and especially highlight the cross-talking between Notch and Wnt signaling pathways in these regeneration processes, shedding light on the improvement of regenerative capacity in vertebrates utilizing these two signaling pathways.

## Notch and Wnt signaling pathways

### Notch signaling pathway

Notch signaling is a highly conserved pathway that mediates cell proliferation, cell fate decision, cell death, and stem cell maintenance in a variety of tissues during development and homeostasis (Schwanbeck et al. 2011). It is also widely involved in various regeneration processes of different organs, including caudal fin, liver, retina, spinal cord, and brain, etc. (Dias et al. 2012; Grotek et al. 2013; Kamei et al. 2012; Wan and Goldman 2017; Zhang et al. 2018).

**Fig. 1:**
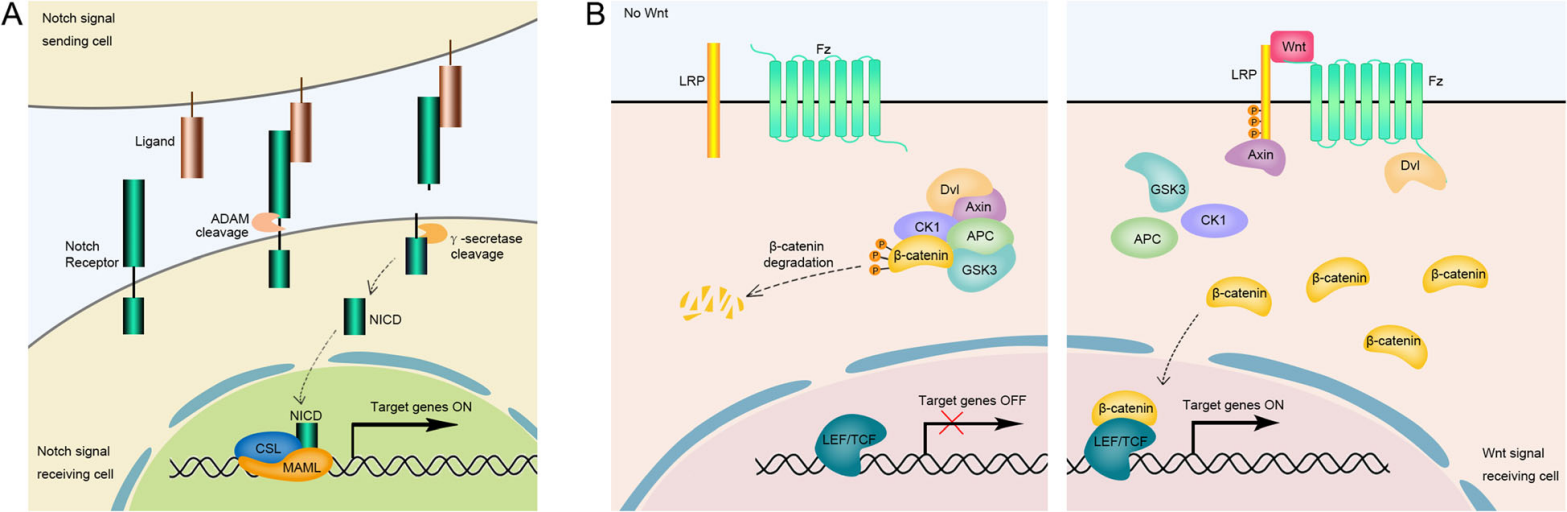
The diagrams of Notch and Wnt pathways. **a** The Notch receptor and ligand are localized at the plasma membranes of neighboring cells. Their binding triggers the sequential cleavage of Notch receptor by ADAM family of metalloproteases and γ-secretase, leading to the release of Notch intracellular domain (NICD), which translocates into the nucleus and facilitates the CSL (CBF1/Su(H)/LAG1) transcription factor, along with the coactivator Mastermind-like (MAML), to activate the transcription of target genes. **b** Without Wnt ligand, β-catenin is hyperphosphorylated within a cytoplasmic destruction complex including Dishevelled (Dvl), Axin, casein kinase 1 (CK1), Adenomatous Polyposis Coli (APC) and glycogen synthase kinase 3 (GSK3), and then degraded via the ubiquitin/proteasome pathway. When Wnt ligand binds to a seven-pass transmembrane receptor Frizzled (Fz) and a single-pass transmembrane co-receptor low-density lipoprotein receptor-related protein (LRP), the Dvl/Axin/CK1/APC/GSK3 complex is dissociated, allowing β-catenin to accumulate in the cytoplasm and subsequently translocate into the nucleus, where β-catenin interacts with transcriptional factors, such as lymphoid enhancer-binding factor1/T cell-specific transcription factor (LEF/TCF), to induce the expression of target genes

The Notch pathway conducts intercellular communications between two neighboring cells, and obtained its name from the Notch receptor, a single-pass transmembrane protein (Gridley 2010; Okuyama et al. 2008). In mammals, four Notch receptors (Notch1–4) are known, containing both extracellular and intracellular domains. At the plasma membrane, Notch protein is activated by a DSL (Delta/Serrate/LAG-2) family of ligand, which is known as the Delta-like-type ligands DLL1/3/4 or Jagged-type ligands Jag1/2 in mammals, and sequentially cleaved by ADAM family of metalloproteases complex and γ-secretase, leading to the release of Notch intracellular domain (NICD). In the canonical Notch pathway, NICD translocates into the nucleus and binds to the CSL (CBF1/Su(H)/LAG1) transcription factor, known as RBP-jκ in mammals (Klüppel and Wrana 2005). Eventually, the CSL transcription factor, along with the coactivator Mastermind-like (MAML), activates the transcription of target genes, including *Hairy enhancer of split* (*Hes)* and *Hes related to YRPW motif (Hey)* families (Watt et al. 2008). In brief, the canonical Notch signaling pathway refers to a NICD-CSL-MAML signal transduction cascade (Fig. 1a).

### Wnt signaling pathway

Wnt signaling regulates cell fate determination, cell proliferation, and cell polarity in the development of all metazoans (Ling et al. 2009; Pond et al. 2020; Wen et al. 2020). In the canonical pathway, Wnt ligand binds to a seven-pass transmembrane receptor Frizzled (Fz) and a single-pass transmembrane co-receptor low-density lipoprotein receptor-related protein (LRP), activating the downstream signal transduction involving Dishevelled (Dvl), glycogen synthase kinase 3β (GSK3β), casein kinase 1 (CK1), Axin, Adenomatous Polyposis Coli (APC) and β-catenin (Fig. 1b). These proteins act as integral transcription regulators in the canonical Wnt signaling pathway. Without Wnt ligand, β-catenin is hyperphosphorylated within a cytoplasmic destruction complex including Axin, CK1, APC, and GSK3β, and then degraded via the E3 ubiquitin ligase β-TrCP-mediated ubiquitin/proteasome pathway. When the Wnt-Fz binding initiates the pathway, phosphorylation of Dvl promotes the dissociation of Axin/CK1/APC/GSK3β complex, allowing β-catenin to accumulate in the cytoplasm and subsequently translocate into the nucleus, where β-catenin interacts with transcriptional factors, such as lymphoid enhancer-binding factor1/T cell-specific transcription factor (LEF/TCF), to induce the expression of target genes (Angers and Moon 2009). The non-canonical Wnt pathway, such as the planar cell polarity (PCP) and Wnt-calcium signaling, are transduced from Fz receptor to alternative intracellular messengers and effectors independent of β-catenin (Angers and Moon 2009; Gao and Chen 2010).

### Interaction between Notch and Wnt signaling pathways

The complete pathway of Notch or Wnt signaling is found in all multicellular animals, including poriferans, but not in fungi or protozoans (Gazave et al. 2009; Holstein 2012). When these two pathways are simultaneously active, there must be certain cross-talks between them to coordinate and fine-tune their actions (Borggrefe et al. 2016; Collu et al. 2014). Indeed, the Notch receptor has been reported to negatively regulate the stability of β-catenin (Acosta et al. 2011; Kwon et al. 2011). Conversely, the gene encoding Jag1, a Notch ligand, is a target of canonical Wnt/β-catenin signaling (Estrach et al. 2006; Katoh and Katoh 2006a; Pannequin et al. 2009). Notch target gene *Hes1* is also regulated by Wnt/β-catenin signaling at the transcription level (Li et al. 2012; Shimizu et al. 2008). Numb, an inhibitor of Notch signaling, is a potent target of the Wnt/β-catenin signaling in multiple types of progenitor cells (Katoh and Katoh 2006b). Moreover, GSK3β, a component in the destruction complex mediating β-catenin degradation, can phosphorylate NICD, which in turn promotes NICD nucleus localization and increases its transcriptional activity and stability (Collu et al. 2014). Dvl is able to bind with NICD to trigger its endocytosis and degradation (Muñoz-Descalzo et al. 2014), or bind with the CSL transcription factor of Notch pathway and inhibit its transcriptional activity (Collu et al. 2012). All evidence above has indicated that Notch and Wnt signaling pathways synergistically or counteractively interact with each other at multiple levels of signal transduction cascade.

## Roles of Notch and Wnt pathways in regeneration

Organ or tissue regeneration and repair often involve the re-activation of developmental signaling. As the classic developmental signals, Notch and Wnt signaling pathways have been involved in diverse regenerative processes (Atkinson et al. 2015; Wilson and Radtke 2006). Intriguingly, the interaction between the Notch and Wnt pathways has been evidenced by many regeneration studies in ear, bone, heart, liver, and other organ/tissues.

### Hair cell regeneration in the cochlea

The mammalian inner ear can be subdivided into a vestibular system dorsally and the cochlea ventrally, which are responsible for body balance and hearing, respectively (Atkinson et al. 2015; Groves and Fekete 2012). The sensory hair cells (HCs) in the cochlea connect with neurons to convey mechanical sound information into neural impulses. In mammals, only neonatal cochleae have a limited degree of HC regeneration after damage (Bramhall et al. 2014; Cox et al. 2014; Hu et al. 2016), and this regenerative capacity diminishes soon in postnatal mammals (Cox et al. 2014; Maass et al. 2015). The mature cochlea completely lacks the ability to replace damaged HCs spontaneously. The mammalian vestibular sensory epithelium, which uses its hair cells for sensing balance and motion, has low regenerative capacity in adults (Lin et al. 2011), which is not sufficient to fully recover vestibular function after injury. Thus, in mammals, the damage of HCs typically leads to the formation of sensory epithelial scars and irreversible hearing loss and balance deficits. In contrast, in non-mammalian vertebrates, HCs in both auditory and vestibular systems constantly renew and regenerate after injury, restoring the hearing and balance function throughout the life (Balak et al. 1990; Stone and Cotanche 2007). During HC regeneration, the cell source to regenerate new HCs is the non-sensory supporting cell (SC) populations, which normally surround the HCs and function to provide the protection and structural support for HCs in the cochlea (Cox et al. 2014; White et al. 2006).

In newborn mouse ears, Wnt proteins and Wnt pathway components are upregulated after HC damage, and pharmacological inhibition of Wnt signaling decreases the spontaneous HC regeneration in these ears (Hu et al. 2016), indicating a requirement of Wnt signaling for the HC regeneration process. Leucine rich repeat G-coupled receptor 5 (Lgr5) is a downstream target of canonical Wnt signaling and labels actively dividing stem cells/progenitor cells across a diverse range of tissue (Barker et al. 2007; de Lau et al. 2011; Jaks et al. 2008; Kemper et al. 2012). It has been demonstrated that the Lgr5-expressing progenitor cells possess the ability of self-renewal, proliferation, and regeneration into HCs (Bramhall et al. 2014; Cox et al. 2014; Wang et al. 2015). Treatment of Lgr5+ progenitor cells in the neonatal cochlea with Wnt agonists induces cell proliferation and differentiation into HCs, whereas Wnt antagonists reduce their proliferation (Chai et al. 2012; Shi et al. 2013; Shi et al. 2012). Recently, more thorough function and lineage analysis have defined more distinct SC subpopulations (McGovern et al. 2019; Zhang et al. 2019). A population of SCs with Frizzled-9 (Fz9) expression was identified as the progenitors for the generation of HCs in the neonatal mouse cochlea. These Fz9+ cells possess a similar capacity for proliferation, differentiation, and HC generation as Lgr5+ progenitors, but they are a much smaller cell population than Lgr5+ progenitors (Zhang et al. 2019). Given that Fz9 is a receptor of Wnt, this study supports again the importance of Wnt signaling in defining the cell population of progenitors for HC regeneration in the neonatal cochlea. Different from the neonatal cochlea, the adult cochleae do not proliferate and regenerate in response to Wnt signaling, possibly due to the decreased expression of Lgr5 in the adult (Shi et al. 2013). In non-mammalian vertebrates, which can spontaneously regenerate sensory HCs, the effect of active canonical Wnt signaling in promoting HC proliferation and regeneration is more robust. In zebrafish lateral line or chicken auditory organ (basilar papilla), Wnt activation enhances the generation of sensory HCs, whereas Wnt inhibition suppresses the proliferation and regeneration of sensory HCs (Head et al. 2013; Jacques et al. 2014; Jiang et al. 2014; Li et al. 2017; Romero-Carvajal et al. 2015). In summary, Wnt/β-catenin signaling promotes HC regeneration in the mammalian neonatal cochlea and non-mammalian vertebrates as well.

Notch signaling is known as a fundamental pathway to regulate the cell fate determination and mosaic pattern formation of HC and SC during the inner ear development (Li et al. 2015; Romero-Carvajal et al. 2015). In the neonatal mice cochlea, which has the ability to generate HC, expression of Notch target genes, *Hes1*, *Hes5*, *Hey1*, *HeyL*, and *Jag1* are decreased upon HC damage (McGovern et al. 2018). Similar down-regulation of Notch pathway gene expressions were revealed in transcriptomic analyses of zebrafish lateral line and chicken cochleae after damage (Jiang et al. 2014; Jiang et al. 2018). These results suggest that the down-regulation of Notch signaling might be required to initiate HC regeneration after injury. Consistently, in the neonatal mice cochlea with HC loss, the spontaneous HC regeneration can be prevented by increased Notch signaling in SCs (McGovern et al. 2018). The inhibition of Notch signaling by either knocking out (KO) Notch or using the γ-secretase inhibitors, which prevent Notch receptor cleavage and subsequent nuclear translocation of NICD, is able to enhance the SC proliferation and their trans-differentiation into HCs (Hu et al. 2016; Korrapati et al. 2013; Li et al. 2015). In zebrafish lateral line and chicken basilar papilla, inhibition of Notch signaling with the γ-secretase inhibitor DAPT causes excessive regeneration of HCs after damage (Daudet et al. 2009; Ma et al. 2008). It has been reported that inhibition of Notch signaling upregulates the expression of gene *Atoh1* (Itoh and Chitnis 2001; Mizutari et al. 2013; Yamamoto et al. 2006). Atoh1 is a key transcription factor to determine the HC cell fate, and overexpression of *Atoh1* is able to induce the SC-to-HC trans-differentiation in mammalian inner ear (Zheng and Gao 2000). Additionally, Notch signaling positively regulates the expression of cell-cycle inhibitor *cdkn1bb* (Romero-Carvajal et al. 2015). Together, Notch signaling may function to maintain the SCs in a quiescent status and thus block HC regeneration through inhibiting *Atoh1*-mediated SC-to-HC trans-differentiation and/or limiting the cell cycle reentry of SCs.

The expression of Notch pathway genes in the mouse cochlea declines rapidly during the first postnatal week, and this down-regulation persists in adulthood. Therefore, in contrast to the neonatal cochlea, the adult cochlea entirely loses the ability to respond to a Notch pathway blockade (Maass et al. 2015). Consistently, a transient activation of Notch signaling together with Myc overexpression is required to trigger the reprogramming of adult SCs, which then respond to the induction signal by Atoh1 to transdifferentiate into HCs (Shu et al. 2019). All these results imply that adult SCs may lose some properties, such as Notch signaling activity, compared to young SCs, resulting in the inability to regenerate HCs. However, it has been shown that the γ-secretase inhibitors are able to stimulate cochlear HC regeneration and partial recovery of hearing ability in the damaged model of adult cochlea (Mizutari et al. 2013; Tona et al. 2014). These controversial results could be explained as a sub-population of SCs with significant Notch activity exists in the adult cochlea and transdifferentiates into HCs in response to the γ-secretase inhibitor-mediated Notch inhibition. It is also possible that the γ-secretase inhibitor functions on another target, which is Notch-independent but also able to affect SC-to-HC trans-differentiation.

**Fig. 2:**
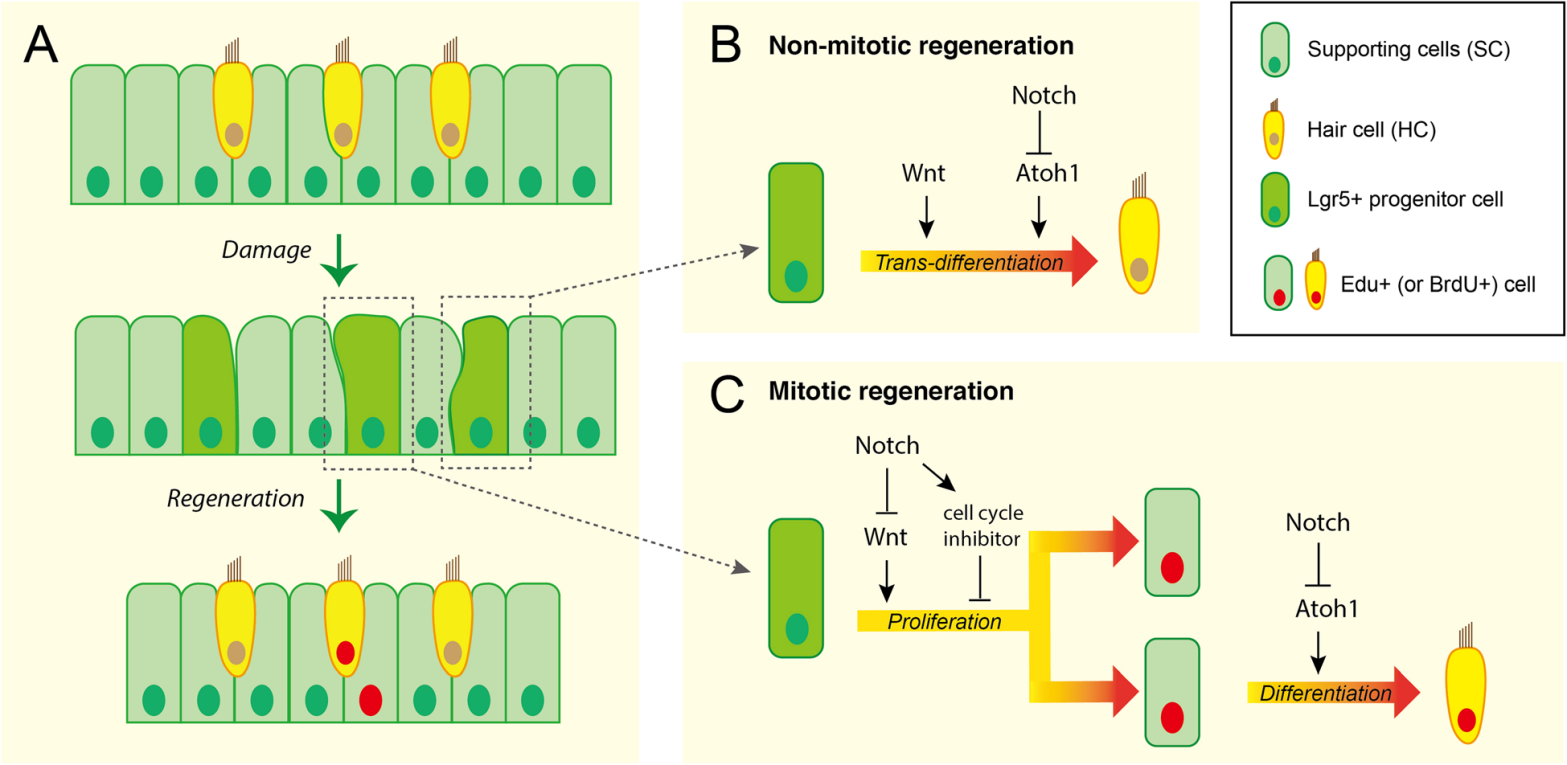
The roles of Notch and Wnt signaling in hair cell regeneration. **a** Upon damage, supporting cells (SCs) can give rise to the new hair cells (HCs) through non-mitotic regeneration (**b**) or mitotic regeneration (**c**). **b** SCs directly trans-differentiate into HCs without entering the cell cycle. Wnt signaling promotes this process, whereas Notch signaling inhibits it though Atoh1, a key transcription factor for HC cell fate commitment. **c** During the mitotic regeneration, SCs proliferate first and then differentiate into new HCs. Wnt signaling is able to induce SC proliferation. Notch signaling represses SC proliferation and differentiation via the inhibitions of Wnt signaling and *Atoh1*expression or limiting the cell cycle reentry of SCs. Usually, the mitotic markers, such as Edu or BrdU, are used to indicate the new SCs/HCs from proliferation, which can be distinguished from the non-mitotic regenerated HCs

During HC regeneration, SCs can give rise to the new HCs through either the mitotic regeneration mechanism, in which SCs proliferate first and then differentiate into HCs, or the direct trans-differentiation mechanism, in which SCs directly trans-differentiate into HCs without first entering the cell cycle (Cox et al. 2014) (Fig. 2). Usually, the mitotic markers, such as Edu or BrdU, are used to indicate SC proliferation during mitotic regeneration. In non-mammal vertebrates, SCs can give rise to the new HCs through both the mitotic and non-mitotic mechanism. However, many studies have found that the majority of generated HCs comes from the direct trans-differentiation of SCs in mammalian cochleae (Chai et al. 2012; Cox et al. 2014). Given that direct trans-differentiation of SCs into HCs exhausts the population of SCs, therefore, the HC generation with proliferation is considered as a better choice for the regeneration of HCs to restore hearing. To achieve better efficiency for mitotic HC regeneration, co-regulation of Wnt and Notch signaling pathways were recently investigated.

In neonatal mouse cochleae, either overexpressing β-catenin or deleting Notch1 in SCs is able to significantly increase the SC proliferation. However, very few proliferating SCs were observed in Notch1 and β-catenin double knockout transgenic mice (Ni et al. 2016), implying that the SC proliferation induced by Notch1 deletion is dependent on β-catenin. Moreover, Notch signaling needs to be downregulated to activate Wnt-induced proliferation (Li et al. 2015; Ni et al. 2016). Similarly, in zebrafish lateral line, Wnt pathway genes are upregulated upon downregulation of Notch signaling, and Notch signaling inhibits SC proliferation via inhibition of Wnt (Romero-Carvajal et al. 2015). These results suggest that Notch signaling acts as an upstream and negative regulator of Wnt/β-catenin signaling to inhibit SC proliferation. Furthermore, simultaneously inhibiting Notch and upregulating Wnt/β-catenin in neonatal mouse cochlea and utricles led to more significant enhancement of the SC proliferation than manipulating either pathway alone (Ni et al. 2016; Wu et al. 2016), suggesting a synergistic effect of Notch inhibition and Wnt activation on SC proliferation. However, by combining β-catenin overexpression and Notch1 deletion, the mitotic HC generation in the neonatal cochlea is still very limited (Ni et al. 2016). Considering that Atoh1 acts as a key transcriptional factor during the cell fate determination of HCs, co-activation of β-catenin and Atoh1 in neonatal cochlear Lgr5+ cells is able to increase the proliferation of SCs and their differentiation into HCs (Kuo et al. 2015). Intriguingly, the triple manipulations of Notch1 deletion, β-catenin overexpression, and *Atoh1* overexpression in SCs induce massive SC proliferation and extensive mitotic generation of HCs (Ni et al. 2016). All the above studies suggest that co-manipulation of multiple effectors could be promising approaches to achieve both SC proliferation and differentiation into HCs after HC loss.

### Bone and fin regeneration

#### Bone regeneration

**Fig. 3:**
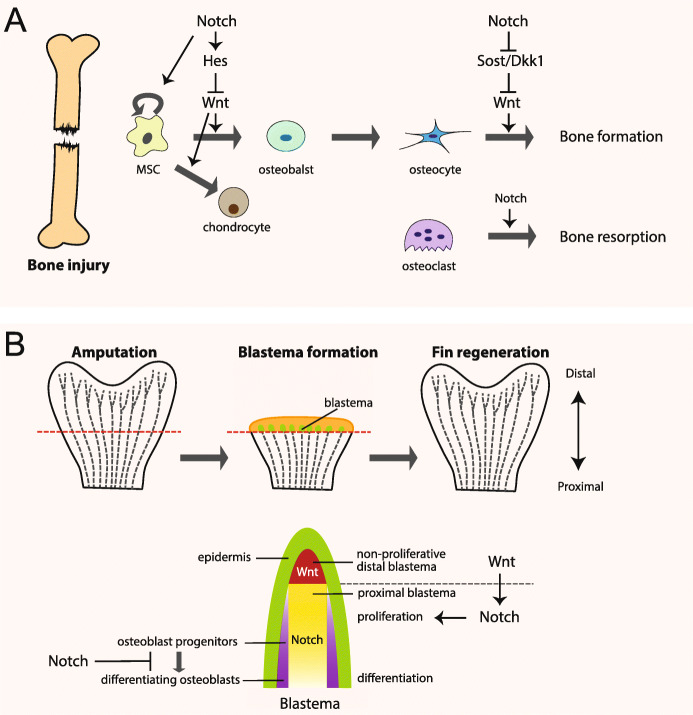
The role of Notch and Wnt signaling in bone or zebrafish fin regeneration. **a** Upon bone injury, the bone marrow mesenchymal stem cells (MSCs) give rise to osteoblasts, which terminally differentiate into osteocytes for bone repair. The osteoblasts, the bone-forming cells, cooperate with the multinucleated osteoclasts, the bone-resorbing cells, to control the bone remodeling. The canonical Wnt/β-catenin signaling enhances bone healing through accelerating MSC differentiation into osteoblasts and chondrocytes, which form cartilage. In contrast, Notch signaling in MSCs functions to inhibit their differentiation through Hes1-mediated suppression of Wnt signaling. In osteocytes, activation of Notch signaling promotes bone formation by inhibiting the expressions of Wnt-inhibitor *Sost* and *Dkk1*. In osteoclasts, Notch signaling is required for bone resorption. **b** During zebrafish tail fin regeneration, a mass of undifferentiated proliferating mesenchymal progenitor-like cells at the amputation plane form the blastema, which gives rise to all the cell types that form the new fin. Notch signaling in proximal blastema functions to maintain blastema cells in an undifferentiated and proliferative state and block osteoblast differentiation. Wnt signaling is required for the blastema formation and subsequent proliferation. Wnt signaling is active in non-proliferative distal blastema and functions upstream of Notch signal to regulate blastema cell proliferation

Bone is a unique tissue that can completely regenerates in all vertebrates, rather than healing with a scar after injury (Dimitriou et al. 2011). Under certain situations like fracture, trauma, and osteoporosis, bone regeneration is required in large quantities. Bone formation and regeneration involve the coordinated response of many types of cells. Osteoblasts, the bone-forming cells derived from bone marrow mesenchymal stem cells (MSCs) can differentiate into osteocytes or die by apoptosis. Osteocytes are terminally differentiated cells embedded in the mineralized matrix. The multinucleated osteoclasts are the bone-resorbing cells. Osteoblasts and osteoclasts cooperate to regulate the modeling of the growing bone, and also controls the bone remodeling throughout life. It has been suggested that both Notch and Wnt signaling pathways as well as their downstream networks are implicated in bone formation and regeneration (Knight and Hankenson 2013) (Fig. 3a).

During development, canonical Wnt/β-catenin signaling has been well established to positively regulate bone formation (Krishnan 2006), through diverse mechanisms, including inducing osteoblastogenesis from MSCs (Bennett et al. 2005), promoting osteoblast proliferation and differentiation (Yan et al. 2009), protecting osteoblasts from apoptosis (Bodine et al. 2005; Bodine et al. 2004), and inhibiting osteoclast formation to suppress bone resorption (Glass et al. 2005). In contrast, Notch signaling maintains bone marrow mesenchymal progenitors by inhibiting MSC differentiation into osteoblasts in the early stage (Hilton et al. 2008; Ji et al. 2017; Tu et al. 2012), but enhances osteoblast differentiation into osteocytes and inhibits osteoclast formation and bone resorption in late stage (Canalis et al. 2013; Engin et al. 2008; Ji et al. 2017; Liu et al. 2016). Moreover, canonical Notch signaling activation in osteocytes decreases osteoclasts and bone resorption, and increases the bone volume (Canalis et al. 2016). Different from the mice model, in human bone marrow mesenchymal stem cells (BMSCs), Notch signaling activation consistently induces osteogenic differentiation. The treatment of human MSCs with Jag1, or overexpression of NICD2, leads to an increase in osteoblast related genes *ALP* and *Bone Sialoprotein*, and a downregulation of TWIST1/2, the negative regulators of osteogenic differentiation, hence an induction of osteoblastogenesis (Osathanon et al. 2019; Zhu et al. 2013).

Upon bone injury, the expression of Wnt ligands, receptors, β-catenin, and signaling reporters are upregulated (Chen et al. 2007; Kim et al. 2007; Leucht et al. 2008). The bone healing was significantly repressed in the mice with an osteoblast-specific null allele of β-catenin, but dramatically enhanced in mice expressing an activated form of β-catenin in osteoblasts (Chen et al. 2007). Similarly, knocking out LRP5 in mice, or overexpressing DKK1, a Wnt antagonist that binds to LRP5, inhibits the healing process (Chen et al. 2007; Komatsu et al. 2010), whereas the repression of DKK1 or treatment with a GSK3β inhibitor improves bone repair and regeneration (Komatsu et al. 2010; Li et al. 2011b; Sisask et al. 2013). Furthermore, Wnt3a injection into the periosteum induces faster bone regeneration by stimulating the proliferation of skeletal progenitor cells and accelerating their differentiation into osteoblasts (Minear et al. 2010). In addition to osteocyte lineage, inhibiting Wnt/β-catenin signaling in chondrocytes also compromises fracture healing due to reduced and delayed cartilage formation and bone generation in mice (Huang et al. 2012). Accordingly, a population of long-lived skeletal cells on the periosteum of a long bone with Axin2 gene expression was identified as Wnt-responding cells, which are activated upon injury and give rise to both cartilage and bone for repair (Ransom et al. 2016). All these results support a positive role of Wnt signaling for bone regeneration.

Notch signaling is also playing important roles during bone regeneration and healing. The expressions of multiple genes in the canonical Notch pathway are upregulated, such as *Jag1*, *Notch3*, *Hes1*, and *Hey1*, during tibial fracture healing (Dishowitz et al. 2012; Osathanon et al. 2019). The transgenic mice with repressed Notch signaling by expressing a dominant-negative form of MAML (dnMAML) shows abnormal bone maturation and remodeling after injury (Dishowitz et al. 2013). Further cell-lineage specific studies have revealed that the role of Notch signaling seems to be complicated during bone healing. Loss of Notch signaling in BMSCs by deleting *RBP-jκ* results in fracture nonunion likely due to the defective BMSC pool (Wang et al. 2016), suggesting that Notch signaling may function to maintain MSC at a proliferative status and thus inhibit MSC differentiation. Accordingly, for bone repair, Notch signaling needs to be repressed to allow MSC differentiation. Indeed, downregulation of Notch signaling has been observed after fracture in a particular population of MSCs with alpha smooth muscle actin (αSMA) marker, which contributes to osteochondral elements during fracture healing (Matthews et al. 2014). In contrast, in osteoblasts or chondrocytes, the removal of Notch signaling has no effects on the fracture repair process (Wang et al. 2016). In osteocytes, activation of Notch signaling promotes bone healing following osteotomy (Liu et al. 2016). Suppression of Notch signaling in osteoclasts by dnMAML reduces osteoclastic resorption and improves bone regeneration and healing (Goel et al. 2019). Collectively, Notch signaling regulates bone regeneration in a cell-context dependent manner, and the underlying molecular mechanism in details needs to be further investigated.

The crosstalk between Notch and Wnt signaling for bone formation has been implicated in several studies. An in vitro study has demonstrated that NICD overexpression in MSCs impairs osteoblastogenesis through suppressing Wnt/β-catenin signaling mediated by Hes1 (Deregowski et al. 2006). Activation of canonical Notch signaling in osteocytes increases bone formation by inhibiting *Sost* and *Dkk1* expressions and consequent upregulation of Wnt signaling, which effects are disappeared in the context of the *RBP-jκ* inactivation (Canalis et al. 2016). Furthermore, the interaction between Notch and Wnt signaling has been considered in the therapeutic treatment for bone healing. It was reported that medicarpin, a natural pterocarpan, promoted bone regeneration and healing by activating canonical Notch and Wnt signaling pathways (Dixit et al. 2015).

#### Zebrafish fin regeneration

The zebrafish caudal fin is a complex structure, with 16–18 segmented bony fin rays (lepidotrichia) that are directly formed from osteoblasts, and the soft interray tissue. Each bony fin ray is formed by two concave hemirays, which are lined with osteoblasts on surfaces and serve to protect a core of blood vessels, nerves, melanocytes, fibroblasts, and mesenchymal cells (Tal et al. 2010). The caudal fin provides a productive model of limb regeneration and bone repair because it is easily accessible and not essential for survival (Akimenko et al. 2003; Poss et al. 2003). Complete caudal fin regeneration takes around 14 days and comprises three phases: wound healing, blastema formation, and regenerative outgrowth (Munch et al. 2013). Several signaling pathways have been found to be required for fin regeneration, including Notch and Wnt pathways (Poss 2010; Stoick-Cooper et al. 2007; Tal et al. 2010) (Fig. 3b).

During zebrafish fin regeneration, the tissue blastema, a mass of undifferentiated proliferating mesenchymal progenitor-like cells at the amputation plane, gives rise to all the cell types that form the new fin. It has been demonstrated that the blastemal cells are partially derived from dedifferentiated mature osteoblasts (Knopf et al. 2011). Notch signaling is activated early in the blastema and remains active throughout the regeneration process. It functions to maintain blastema cells in an undifferentiated and proliferative state during fin regeneration. Notch signaling inhibition with inhibitors or morpholinos (MO) reduces blastema cell proliferation and impairs fin regeneration. Overexpression of NICD in the regenerating fin leads to the expansion of blastema, but the reduction of osteoblast differentiation, and thus inhibition of bone regeneration (Grotek et al. 2013; Munch et al. 2013). Wnt/β-catenin signaling is activated in distal blastema during zebrafish tail fin regeneration (Stoick-Cooper et al. 2007; Wehner et al. 2014). The studies from Wnt inhibitor Dkk transgenic fish revealed that Wnt/β-catenin signaling is required for the blastema formation and subsequent proliferation of the blastema (Stoick-Cooper et al. 2007). Wnt10a is possible the primary Wnt ligand responsible for early activation of this pathway during fin regeneration. Enhanced Wnt/β-catenin signaling by overexpressing Wnt ligand or GSK3β inhibitor treatment is sufficient to induce faster regeneration by elevating cell proliferation and osteoblast differentiation in fins (Sarmah et al. 2019; Stoick-Cooper et al. 2007). Moreover, the analysis of gene expression profile during regeneration revealed that Wnt/β-catenin signaling can regulate multiple key signals, including Notch. The expression of Notch signaling target genes, *her* family, can be altered upon Wnt inhibition, whereas Notch inhibition has little effect on Wnt signaling activity (Wehner et al. 2014). Therefore, Wnt/β-catenin signaling function upstream of Notch signal to orchestrate growth and differentiation of the regenerating fin.

### Heart regeneration

As the most important organ of vertebrates, the damage of the heart is fatal for individuals. Myocardial infarction (MI) is a leading cause of morbidity and mortality globally, which are characterized by the irreversible loss of cardiomyocytes and replacement with fibrosis scar, increasing susceptibility to heart failure and sudden death. So the regenerative capacity of the heart is beneficial for survival, however, it is actually greatly variable among species. The heart of adult mammals, including humans, fails to recover structurally or functionally after injury, owing to a permanent scar deposition of massive fibrotic tissue, along with an extremely low renewal rate of cardiomyocytes (Bergmann et al. 2009; Bergmann et al. 2015). However, neonatal mammals possess a certain capacity to regenerate heart tissue. The heart in neonatal mouse or neonatal pig exhibits a transient regenerative potential, which is dampened quickly during the first week or 2 days of postnatal life, respectively (Porrello et al. 2011; Ye et al. 2018; Zhu et al. 2018). Strikingly, adult zebrafish can completely regenerate their hearts in 30–120 days in different cardiac injury models, such as apical resection, ventricular cryoinjury or genetic ablation of cardiomyocytes (Chablais et al. 2011; Gonzalez-Rosa et al. 2017; Gonzalez-Rosa and Mercader 2012; Poss et al. 2002; Wang et al. 2011). Different from a permanent scar formation in adult mammals, the scar in the fish heart is eventually dissolved and the injured tissue is replaced by new cardiomyocytes (Gonzalez-Rosa et al. 2011). The natural capacity for cardiac regeneration exhibited by adult zebrafish and neonatal mammalian suggests the possibility that adult mammal hearts could be stimulated to regenerate if the cellular and genetic determinants or signaling pathways for cardiomyocyte proliferation were fully elucidated.

Notch signaling pathway plays pivotal roles during heart development and disease processes, reviewed recently (Luxan et al. 2016). Current studies have characterized the potential benefits of Notch activation for reducing infarct size and improving cardiac function after myocardial infarct in mice (Gude et al. 2008) and the involvements of Notch pathways during heart regeneration in zebrafish (Munch et al. 2017; Raya et al. 2003; Zhao et al. 2014). During zebrafish heart regeneration, Notch signaling is activated after cardiac injury in either ventricular resection or cryoinjury models, including the elevated gene expressions of Notch receptors (*notch1a, notch1b*, *notch2*, *and notch3*), ligands (*dlc* and *dll4*), and the signaling modulator lunatic fringe (*lfng*) (Munch et al. 2017; Raya et al. 2003; Zhao et al. 2014). Suppression of Notch pathway through either transgenic expressing Notch inhibitory factor DN-MAML or pharmacological treatment with Notch inhibitor RO492909 decreases the cardiomyocyte proliferation rate, impairs the regeneration of new muscle, and induces scar formation at the site of injury (Munch et al. 2017; Zhao et al. 2014). Moreover, ubiquitous Notch pathway activation also compromises zebrafish cardiomyocyte proliferation and cardiac regeneration, indicating that cardiomyocyte proliferative renewal is exquisitely sensitive to perturbations in Notch signaling (Zhao et al. 2014).

**Fig. 4:**
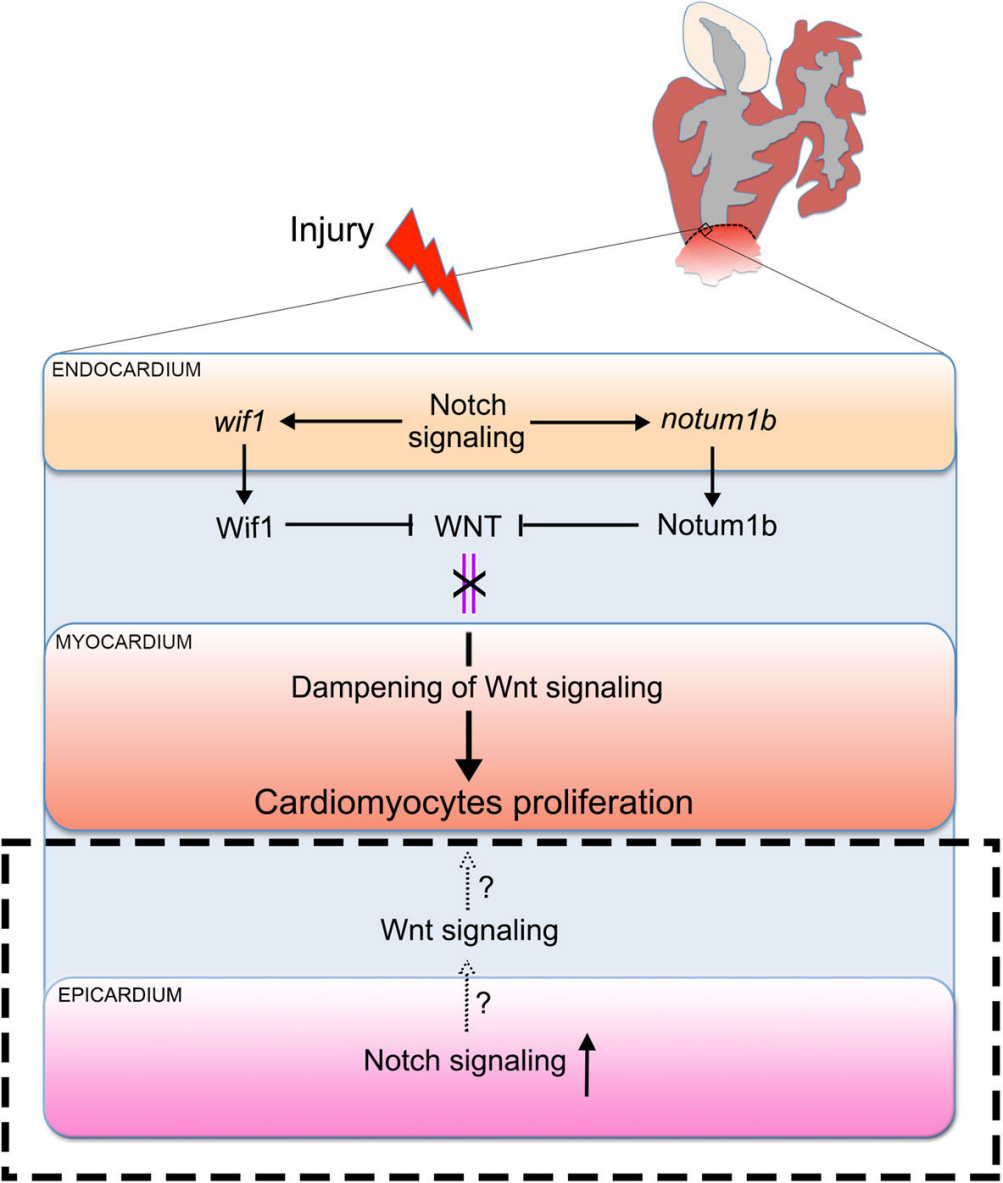
The roles of Notch and Wnt signaling in zebrafish heart regeneration. During zebrafish heart regeneration, Notch signaling functions for CM proliferation and heart regeneration through a paracrine mechanism. After the amputation injury of zebrafish heart, Notch signaling is activated specifically in the endocardium and epicardium, but not in myocardial cells. Endocardial Notch signaling restrains myocardial Wnt pathway activation through inducing the expression of *wif1* and *notum1b*, which encode two secreted Wnt antagonists, therefore promotes cardiomyocyte proliferation in the myocardium. The activated Notch signaling in epicardium may function in a similar manner to facilitate heart regeneration, which requires further exploration in the future

We have reported that the expressions of Notch receptors (*notch1a, notch1b,* and *notch2*) are stimulated specifically in the endocardium and epicardium, but not in myocardial cells, after the amputation injury of zebrafish heart (Zhao et al. 2014), indicating that Notch signaling functions for CM proliferation and heart regeneration through a paracrine mechanism. To understand the lineage-specific requirement for Notch signaling in zebrafish heart regeneration, the consequences of endocardial-specific Notch inhibition is evaluated following cardiac apical amputation injury. This manipulation dampens cardiomyocyte proliferation and leads to regenerative failures, implicating a requirement of endocardial Notch signaling for heart regeneration (Zhao et al. 2019a). RNA-seq profiles in hearts revealed that the expressions of two transcripts, *wif1* and *notum1b*, which encode two secreted Wnt antagonists, are reduced in the endocardium and epicardium upon Notch repression during zebrafish heart regeneration (Zhao et al. 2019a) (Fig. 4). In the cardiac development context, the Wnt pathway is required for mesodermal specification of embryonic stem cells but needs to be deactivated for further differentiation of cardiomyocytes (Ozhan and Weidinger 2015). In adult mice that lacks the capacity of heart regeneration, multiple Wnt pathway components are activated in response to the cardiac injury (Aisagbonhi et al. 2011; Duan et al. 2012), which could be associated with the pathological healing process (Haybar et al. 2019). During zebrafish heart regeneration, several small molecules as Wnt inhibitors positively promote cardiomyocytes proliferation and heart regeneration (Xie et al. 2020), suggesting that Wnt signaling might be reduced to enable natural heart regeneration. Upon cardiac injury in zebrafish, Wnt signaling activity detected by a transgenic reporter accumulates along the edge of the wound area (Stoick-Cooper et al. 2007). Hyperactivation of canonical Wnt signaling by small molecular activator 6-bromoindirubin-3-oxime (BIO) administration impedes cardiomyocyte proliferation and induces scarring after injury (Zhao et al. 2019a). It has also been noted that the non-canonical Wnt pathway might be required to regulate cardiomyocyte proliferation during zebrafish heart regeneration (Peng et al. 2020). Another recent study demonstrated that the CM-specific downregulation of Lrp6, one Wnt co-receptor, could increase CM proliferation and improve cardiac functions after MI in mice (Wu et al. 2020). Significantly, our study has revealed that Wnt pathway inhibition partially rescues the myocardial proliferation deficit observed in endocardial-specific Notch-suppressed hearts, suggesting that endocardial Notch signaling restrains myocardial Wnt pathway activation during heart regeneration (Zhao et al. 2019a) (Fig. 4). This antagonistic crosstalk between Notch and Wnt pathways also exists in many other biological contexts (Boulter et al. 2012; Kwon et al. 2011; Tian et al. 2015). Nevertheless, whether the antagonistic crosstalk of these two pathways is also required in the epicardium during heart regeneration remains tested. Actually, Wnt/β-catenin signaling plays distinct roles during heart development. In the early developmental stage, the Wnt pathway is required for enhancing CM formation, possibly via promoting precursor cell proliferation. However, in the later developmental stage, Wnt activity needs to be repressed for CM differentiation. During regeneration or response to cardiac injury, current studies indicated the converse roles. Further efforts are expected to elucidate the molecular mechanisms underlying the different functions of Wnt signaling in the context of cardiac development and regeneration.

### Liver regeneration

The liver is mainly associated with metabolism in vertebrates and plays essential roles in detoxifying various metabolites, regulating glucose and lipid metabolism, synthesizing serum proteins, and secreting bile. Hepatocytes and cholangiocytes are two major cell types in the liver (Michalopoulos and Bhushan 2020). Hepatocytes conduct most of the hepatic functions and account for more than 80% of liver mass. Cholangiocytes are biliary epithelial cells forming the biliary network that transports bile from hepatocytes to the gallbladder.

**Fig. 5:**
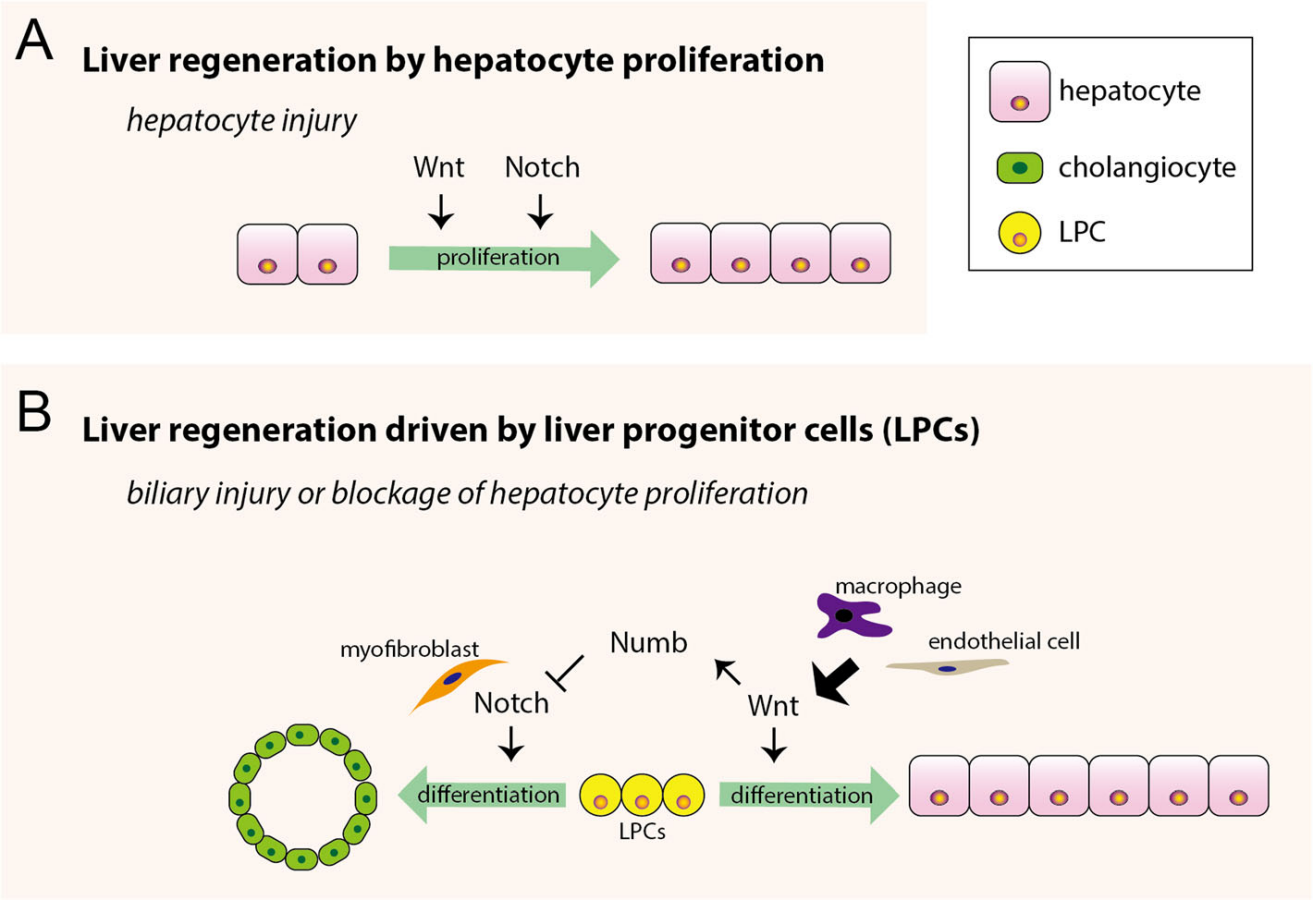
The roles of Notch and Wnt signaling in liver regeneration. **a** In hepatocyte injury models, such as the partial removal of the liver (partial hepatectomy), the liver regeneration is predominantly contributed by the proliferation of existing hepatocytes. Wnt and Notch signaling are required for hepatocyte proliferation and liver regeneration. **b** Following the liver injury with a biliary response or under conditions where the proliferation capacity of the hepatocyte is impaired, liver progenitor cell (LPC)-driven regeneration is an alternative mode to mediate hepatic repair. The myofibroblast-involved Notch signaling activation in LPCs guides their differentiation into cholangiocytes, whereas Wnt proteins secreted from macrophages and endothelial cells promote the differentiation of nearby LPCs into hepatocytes. The Wnt-Notch interaction during this process is mediated by Numb, a direct transcriptional target of canonical Wnt signaling that functions to inhibit Notch signaling

The liver is a highly regenerative organ, and able to restore its mass and function after injury (Michalopoulos and Bhushan 2020). In the context of many injury models, such as the partial removal of the liver (partial hepatectomy), liver regeneration is predominantly contributed by the proliferation/growth of existing hepatocytes (Michalopoulos and Bhushan 2020) (Fig. 5a). Following the liver injury with a biliary response or under conditions where the proliferation capacity of the hepatocyte is impaired, liver progenitor cell (LPC)-driven regeneration is an alternative mode to mediate hepatic repair, in which the biliary-derived or hepatocyte-derived LPCs proliferate and differentiate into cholangiocytes or hepatocytes (So et al. 2020) (Fig. 5b). In addition, it has been suggested that hepatocytes can transdifferentiate into cholangiocytes after certain forms of injuries (Yanger et al. 2013). Although the liver and gallbladder are closely related in position and function, unlike the liver, the gallbladder is not renewable.

In rats, β-catenin migrates rapidly to hepatocyte nuclei within minutes after partial hepatectomy (Monga et al. 2001), suggesting a role of canonical Wnt signaling during the regenerative response of hepatocytes. It has been revealed that Wnt/ β-catenin signaling promotes hepatocyte proliferation for liver regeneration by activating the expression of target genes, such as cell-cycle regulator *cyclin-D1* (Russell and Monga 2018). Eliminating β-catenin in mice can delay liver regeneration but not abolish liver regeneration (Tan et al. 2006; Yang et al. 2014). The Wnt signals are given rise from endothelial cells and macrophages in the damaged liver (Boulter et al. 2012; Preziosi et al. 2018; Zhao et al. 2019b). In response to Wnt signals, rather than a specialized cell population, the hepatocytes throughout the liver can upregulate the expression of the Wnt target gene *Axin2* and contribute to liver regeneration after injury (Sun et al. 2020).

Notch pathway activity increases in hepatocytes following partial hepatectomy in rats and is required for hepatocyte proliferation and regeneration (Köhler et al. 2004; Zhang et al. 2018). In addition, activation of Notch signaling in LPCs in vitro upregulates biliary markers but downregulates hepatocyte markers (Lu et al. 2016). Consistently, during zebrafish liver regeneration, Notch inhibition promotes differentiation of LPCs into hepatocytes, while Notch overactivation impairs this process (Russell et al. 2019). Similarly, regenerative hepatocyte-to-cholangiocyte reprogramming also requires Notch signaling (Yanger et al. 2013). These results indicate that Notch signaling is crucial to commit cholangiocyte fate.

It has been shown that interactions between Notch and Wnt signaling pathways are critical for the fate commitment of LPCs during liver regeneration (Fig. 5b). Jag1 expressed in myofibroblasts activates Notch signaling in LPCs, thereby guiding the differentiation of LPCs into cholangiocytes, whereas Wnt3a secreted from macrophages during liver regeneration suppresses Notch signaling in nearby LPCs, promoting their differentiation into hepatocytes in mice (Boulter et al. 2012). The antagonistic interplay between Notch and Wnt signalings is also revealed during liver regeneration in the ethanol-induced fibrosis zebrafish model, in which a number of Notch antagonists and Wnt agonists were identified through chemical screens to facilitate hepatocyte regeneration in the fibrotic liver of zebrafish. Furthermore, the Wnt-Notch interplay during liver regeneration is mediated by Numb, which is a direct transcriptional target of canonical Wnt signaling but functions to inhibit Notch signaling (Boulter et al. 2012; Huang et al. 2014).

### The regeneration of other organs

**Fig. 6:**
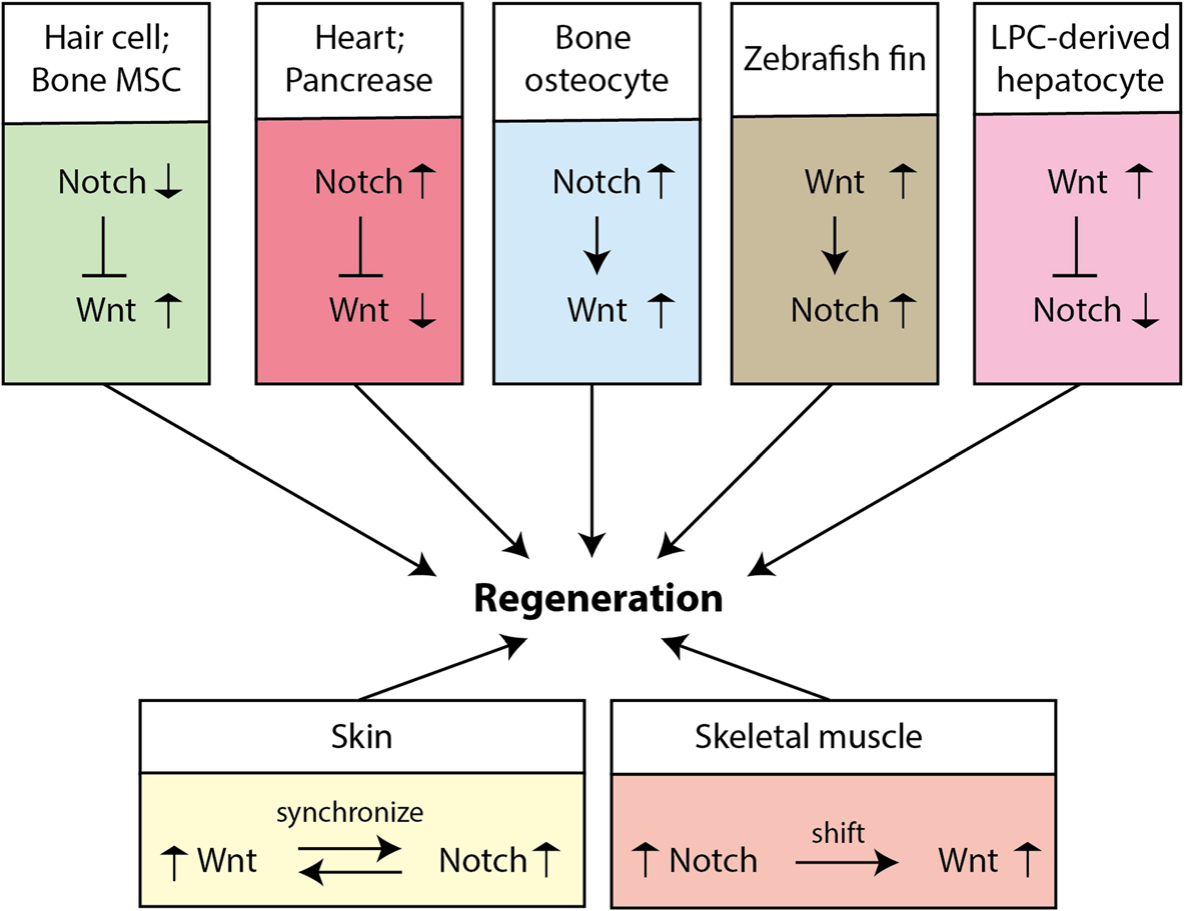
Summary of interaction between Notch and Wnt signaling in representative organ regeneration processes. The relative positions of Notch and Wnt indicate their upstream/downstream relationship in the particular organ regeneration process. The arrows adjacent to the Notch or Wnt are indicating how the signaling activity is altered during regeneration

Skin is the largest organ in the body, including two main layers: the superficial layer, epidermis, and the deeper layer, the dermis. The epidermis functions as a barrier against external microorganisms consisting of a stratified keratinized epithelium interspersed with hair follicles and glands (Martin 1997). Mammalian skin has the remarkable ability to regenerate itself, replacing dead sloughed skin and healing wounds (Adolphe and Wainwright 2005). Wnt/β-catenin and Notch signaling are both crucial mediators of wound repair. Activation of Notch or Wnt/β-catenin signaling promotes wound closure and acquires a thicker epidermis layer, whereas downregulation of Notch or Wnt/β-catenin signaling impairs epidermis re-formation, collagen arrangement, and skin appendage regeneration (Cheon et al. 2002; Chigurupati et al. 2007; Shi et al. 2015). The two pathways might interact in vitro and in vivo. In cultured epidermal stem cells (ESCs) or rat models, the expression levels of Wnt/β-catenin signaling components are significantly elevated in response to Jag1, but decreased after treatment with DAPT, a Notch antagonist. Similarly, the activation of Wnt signaling promotes an upregulated protein expression of Notch components, whereas Wnt inhibition results in downregulated levels of Notch components (Shi et al. 2015). These results imply that Notch and Wnt pathway crosstalk each other to synchronize during skin repair (Fig. 6). Actually, β-catenin can stimulate Notch signaling by inducing *Jag1* transcription during hair follicle formation in adult epidermis (Estrach et al. 2006), which could provide clues to explore the molecular mechanism underlying the Notch and Wnt interaction during skin repair.

Skeletal muscle regeneration in adults is attributed to the presence of satellite cells, which reside between the sarcolemma and the basal lamina in a relatively dormant metabolic state (Yin et al. 2013). Upon injury to skeletal muscle, satellite cells become activated and undergo several rounds of cell division before differentiating into myoblasts, which ultimately fuse with injured myofibers to accomplish regeneration (Yin et al. 2013). Activation of Notch signaling promotes satellite cell self-renewal and proliferation, and inhibits their differentiation into the myogenic lineage through repressing MyoD (Mourikis et al. 2012). By contrast, the activation of canonical Wnt signaling promotes satellite cell differentiation and fusion to injured myofibers (Brack et al. 2008). The temporal switch from progenitor cell proliferation to differentiation is essential for muscle regeneration, which needs a transition from Notch to Wnt signaling in myogenic progenitors (Fig. 6). Interaction between the two pathways occurs via GSK3β, which is maintained in an active form through Notch but is suppressed by Wnt in the canonical Wnt signaling cascade (Brack et al. 2008). Notch/NICD is another component to bridge Wnt and Notch signalings during muscle regeneration. A constitutively phosphoryl-mimicking mutation of Fas-associated death domain (FADD) enhances the phosphorylation of PKCα, which stabilizes Notch-1, resulting in the inhibition of β-catenin accumulation and compromises regeneration of muscles (Zhang et al. 2014).

The regeneration of pancreas also requires Notch signaling, as loss of Notch signaling leads to impaired pancreas regeneration after acute pancreatitis with fewer mature acinar cells. Furthermore, an interaction between Notch and Wnt signaling was identified in pancreatic acinar cells, with NICD1 inhibiting β-catenin-mediated transcriptional activity (Siveke et al. 2008) (Fig. 6).

## Outlook from the view of clinic trials

To date, the attempts of applying Notch and Wnt signaling modulators into the regeneration of human organs or tissues have made significant progress, especially in the regeneration of sensory hair cells. For example, the Notch signaling inhibitor LY3056480 for hair cell regeneration in humans has been clinically tested and shows good clinical effects (Samarajeewa et al. 2019). The combination of Wnt signaling activator CHIR99021 (CHIR) and histone deacetylase inhibitor valproic acid (VPA) can enhance hair cell yield from Lgr5-positive cells isolated from neonatal mice, or adult mice, or non-human primates, or healthy human inner ear tissue (McLean et al. 2017). The drug FX-322, developed as a proprietary combination of CHIR and VPA, has been successfully applied in a first-in-human trial and its safety and tolerability in patients have been confirmed (McLean et al. 2017; Samarajeewa et al. 2019). However, regarding the regeneration of other organs or tissues, clinical trials related to canonical Notch and Wnt signals are still very lacking. Nevertheless, the combination therapy with agents affecting multiple pathways in a human organ or tissue regeneration will be an effective strategy for future clinical trials. With the increasing emphasis and techniques improvement, a more thorough understanding of Notch-Wnt signaling crosstalk and the development of related therapeutic treatments in human organ/tissue regeneration are promising in the near future.

## Conclusions

Organ regeneration recapitulates organ development in numerous aspects, in an attempt to restore the integrity and function of the injured tissue. Obviously, the difference is indeed existing between development and regeneration. The development process is to form an entire organ, whereas regeneration is to generate only the missing part of an organ. Therefore, to achieve the reconstruction of the injury region as the original, the accurate position and pattern information is required for the regenerating cells to determine their locations and cell types, and then a certain mechanism should control the timing to stop the regeneration. In addition, how the newly generated cells are recognized by the pre-existing cells to work as integrity should also be addressed. All of the questions above are of interest in this field, however, still far away from being clearly answered.

Many of the key signaling pathways that are active during development are re-deployed during postnatal tissue repair, including the Notch and Wnt/β-catenin pathways. During development, the canonical Wnt signaling has been well-recognized as a pivotal signal to maintain the stemness of stem/progenitor cells, promote cell proliferation, and guide morphogenesis, while the well-established role of Notch signaling is to determine cell fates. These cellular processes are also involved in the reconstruction of a tissue/organ with full structure and function, so it is not surprising that the Wnt and Notch signaling play important roles during regeneration. It has also been shown that canonical Wnt and Notch pathways genetically interact in many regenerative events in a synergistic or antagonistic manner (Fig. 6). The molecular relationship between these two pathways varies upon different organs and even different cell types. Therefore, the cell-lineage-specific analysis for the Notch-Wnt interaction may provide more valuable information in the future. As the sequential or concurrent manipulation of multiple pathways is likely a more efficient approach to improve the organ regeneration in mammals and humans, better understanding the crosstalk between Notch and Wnt signaling is not only important to deepen our knowledge for regeneration, but also constructive for the clinic attempts. It is noteworthy that non-canonical Notch and Wnt signaling pathways are relatively few studied in the process of organ or tissue regeneration, but we cannot rule out their importance in this process.
